# Enhanced Analysis of Low-Abundance Proteins in Soybean Seeds Using Advanced Mass Spectrometry

**DOI:** 10.3390/ijms26030949

**Published:** 2025-01-23

**Authors:** Bo Meng, Yuanyuan Huang, Ao Lu, Huanyue Liao, Rui Zhai, Xiaoyun Gong, Lianhua Dong, You Jiang, Xinhua Dai, Xiang Fang, Yang Zhao

**Affiliations:** 1 Technology Innovation Center of Mass Spectrometry for State Market Regulation, Center for Advanced Measurement Science, National Institute of Metrology, Beijing 100029, China; meng920816@foxmail.com (B.M.); luao1659097892@163.com (A.L.); liaohuanyue99@163.com (H.L.); zhairui@nim.ac.cn (R.Z.); gxy@nim.ac.cn (X.G.); donglh@nim.ac.cn (L.D.); jiangyou@nim.ac.cn (Y.J.); daixh@nim.ac.cn (X.D.); 2 Department of Grassland Science, College of Forestry and Landscape Architecture, South China Agricultural University, Guangzhou 510642, China; hyy9517@163.com

**Keywords:** soybean seeds, proteomics, BoxCar, DIA, DDA

## Abstract

This study presents an advanced approach for the comprehensive analysis of low-abundance proteins in soybean seeds, addressing challenges posed by high-abundance storage proteins. We compared the effectiveness of Data-Dependent Acquisition (DDA), Data-Independent Acquisition (DIA), and BoxCar mass spectrometry techniques to identify low-abundance proteins in two types of soybean seeds: High-Oil and High-Protein seeds. The results indicate that the DIA method, and particularly the BoxCar methods, significantly improve the detection of low-abundance proteins compared to DDA, offering deeper insights into soybean seed biology. Specifically, BoxCar-based analysis revealed distinct proteomic differences between High-Oil and High-Protein seeds, highlighting more active metabolic processes in High-Oil seeds. Additionally, several key proteins were identified and annotated as uniquely expressed in either High-Oil or High-Protein seeds. These findings emphasize the importance of advanced proteomic techniques, such as BoxCar, in deepening our understanding of soybean seed biology and supporting breeding strategies to improve nutritional qualities.

## 1. Introduction

Soybeans are nutrient-dense legumes containing 18–20% oil and 38–56% plant proteins, making them a crucial global crop for human nutrition and animal feed [1,2]. As the global population grows, the demand for soybeans continues to increase [3]. However, the existing levels of soybean production are insufficient to meet this demand [3]. Enhancing oil and protein yield in soybean seeds has therefore become a critical focus in breeding research. Recent advancements in proteomics have significantly improved our understanding of soybean seed protein composition [4,5,6,7]. Nonetheless, the prevalence of high-abundance proteins presents significant challenges, making the in-depth proteomic analysis of soybean seeds particularly difficult [8,9].

Mass spectrometry (MS) analysis in proteomics typically utilizes two main techniques: Data-Dependent Acquisition (DDA) [10,11,12,13] and Data-Independent Acquisition (DIA) [14,15]. In DDA, MS/MS analysis is performed on the top precursor ions that exhibit the highest intensities in the MS1 scan. Although this approach effectively detects abundant proteins, it may not capture lower-abundance species that could be biologically important. By contrast, DIA acquires MS/MS data on all ions within a specified mass range, enabling the analysis of highly complex samples and improving the detection of low-abundance proteins [16,17,18]. However, this broader coverage can diminish the quality of fragment ion spectra and affect accurate protein identification and quantification.

To overcome these limitations, Mann et al. developed the BoxCar method, which substantially increases dynamic range and detection sensitivity via segmented acquisition [19]. Since its introduction, BoxCar has been applied successfully for the proteomic analysis of samples with high dynamic ranges, such as urine and blood. For instance, Ye et al. used BoxCar to analyze urine samples from healthy individuals and diabetic patients, demonstrating that it detects more low-abundance proteins (thus expanding the dynamic range) and achieves robust reproducibility in identifying these proteins [20]. In another study, Niu et al. employed BoxCar for blood samples from patients with non-alcoholic fatty liver, quantifying 503 proteins across a dynamic range spanning six orders of magnitude [21]. Multiple studies have demonstrated that BoxCar excels at analyzing low-abundance proteins and serves as a critical complement to both DDA and DIA techniques.

Many studies have evaluated the strengths and weaknesses of three primary methods in proteomic studies. For instance, Bekker-Jensen et al. compared DDA and DIA techniques for analyzing the yeast phosphoproteome [22]. They found that DIA identified 1.8 times more phosphorylated peptides and captured ions at a rate 6 times higher than DDA. In another study, Liu et al. explored the plasma proteome and noted that DIA improved protein identification by 41% compared to DDA and significantly reduced the proportion of missing protein identifications from 22% to just 1% [23]. Additionally, Ye et al. investigated the BoxCar and DDA methods using urine samples from both healthy individuals and diabetes patients, demonstrating that BoxCar increased protein and peptide identification by 24.8% and 16.5%, respectively, and greatly improved the detection of low-abundance proteins [20]. Mehta et al. further advanced the field by combining BoxCar and DIA technologies to develop the BoxCarDIA acquisition method, showing that the library-free BoxCarDIA approach outperforms both DDA and directDIA methods with HeLa and Arabidopsis cell samples. Notably, BoxCarDIA achieved a 40% boost in protein quantification compared to DDA, without requiring offline fractionation or additional mass spectrometer acquisition time [24]. Despite these advancements, no systematic evaluation has yet offered a comprehensive comparison of all three methods. Moreover, the differences among these techniques in analyzing the soybean proteome remain largely unexplored.

In this study, we utilized two distinct types of soybean seeds, High Oil and High Protein, to systematically compare the DDA, DIA, and BoxCar methods. Our goal was to determine the most suitable technique for proteomic analyses of soybean seeds. Each method was assessed based on its depth of proteome coverage, reproducibility of protein identification, and ability to detect low-abundance proteins. The results indicated that BoxCar provided the best performance for constructing proteome maps of soybean seeds. By employing this method, we successfully generated and analyzed differential expression proteome maps for both High-Oil and High-Protein soybean seeds, enabling an in-depth investigation of their essential proteins.

## 2. Results

### 2.1. Evaluating Proteomic Methods: DDA, DIA, and BoxCar Workflow

**Figure 1:**
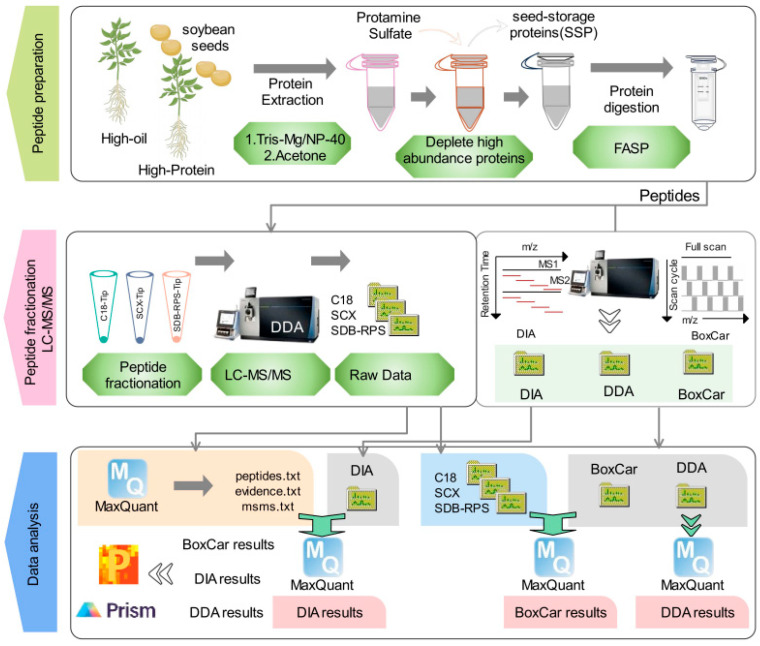
Workflow for DDA, DIA, and BoxCar proteomic methods applied to soybean seeds. The upper panel outlines the initial steps, starting with the removal of high-abundance proteins from soybean seeds using protamine sulfate, followed by protein extraction and peptide generation from the treated seeds. In the middle panel, the left side details the construction of a soybean-specific spectral library, which is essential for subsequent proteomic analyses. The right side illustrates the distinct processes involved in the three proteomic methods: DDA, DIA, and BoxCar. The lower panel focuses on the analysis of raw mass spectrometry data using MaxQuant software, highlighting the distinct processing of spectral data by each method (DDA, DIA, and BoxCar) to achieve comprehensive protein identification and quantification.

Based on data from the China Seed Industry Big Data Platform (http://202.127.42.145/bigdataNew/home/index, accessed on 10 January 2023), Huaxia-2 (HX2) seeds were designated as High-Oil seeds because they contain 21.9% oil and 41.7% protein, whereas Huaxia-14 (HX14) seeds, which contain 18.35% oil and 45.40% protein, were classified as High-Protein seeds. To systematically evaluate the DDA, DIA, and BoxCar methods for soybean seed proteomic analyses, we applied all three to investigate the proteomic differences between HX2 and HX14 seed types (Figure 1).

Proteins were extracted from both High-Oil and High-Protein seeds, and high-abundance storage proteins were removed using protamine sulfate precipitation. The resulting peptides, prepared by the Filter-Aided Sample Preparation (FASP) method [25,26], were divided into two batches. One batch was used for constructing a soybean-specific MS spectral library to support DIA and BoxCar analyses, while the other batch was used directly for DDA, DIA, and BoxCar runs (Tables S1–S3).

During spectral library construction, the peptides were fractionated using three tip-based methods, including C18-Tip [27,28], SCX-Tip (Strong Cation Exchange) [29], and SDB-RPS-Tip (Styrene-Divinylbenzene Reverse Phase Sulfonate) [15], to enhance peptide coverage and improve library robustness. This library enabled precise protein identification and quantification for DIA and BoxCar. DIA utilized the library for efficient high-throughput quantification, while BoxCar leveraged it for an improved dynamic range and sensitivity. To ensure reproducibility, each method was repeated five times, refining data accuracy and minimizing variability.

### 2.2. Spectral Library Construction for Soybean Proteomic Analysis

**Figure 2:**
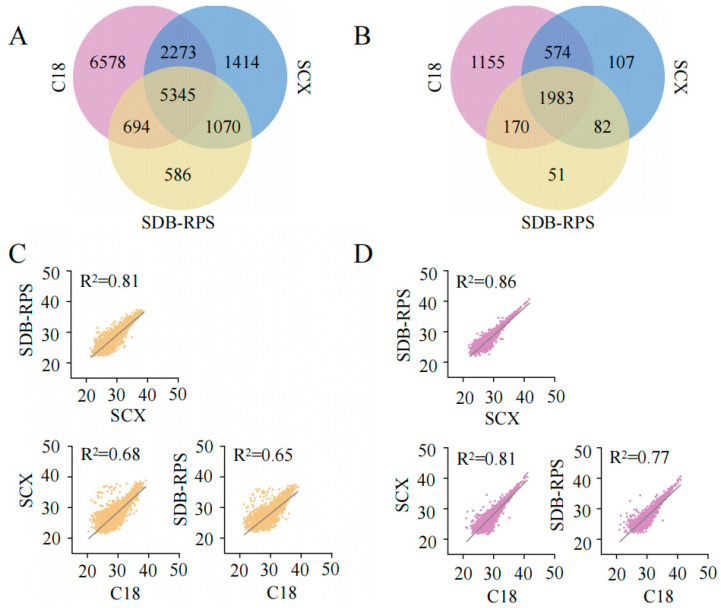
A comparative analysis of the C18-Tip, SCX-Tip, and SDB-RPS-Tip methods. (**A**) A Venn diagram showing the overlap of peptide identifications among the C18-Tip (pink), SCX-Tip (blue), and SDB-RPS-Tip (yellow) methods, highlighting unique and shared peptides identified by each technique. (**B**) A Venn diagram illustrating the overlap of protein identifications between the three methods, demonstrating common and unique proteins detected by each method. (**C**,**D**) Linear correlation analyses of peptide ((**C**) yellow and protein (**D**) pink) abundance identifications between the methods: C18-Tip vs. SCX-Tip, C18-Tip vs. SDB-RPS-Tip, and SCX-Tip vs. SDB-RPS-Tip.

High-abundance storage proteins were removed from soybean seeds using protamine sulfate treatment [30], after which proteins were enzymatically digested using the FASP method [24]. The resultant peptides were pooled in equal amounts and fractionated using C18-Tip, SCX-Tip, and SDB-RPS-Tip (Figure 1). The fractionated samples were subjected to DDA to generate a comprehensive spectral library for DIA and BoxCar analyses. The library, processed with MaxQuant [30,31], identified 17,960 peptides and 4122 proteins (Table S4). Among these, 5345 peptides were consistently detected across all fractionation methods, comprising 29.76% of the total (Figure 2A). Notably, the C18-Tip method uniquely identified 6578 peptides (36.63% of the total), while SCX-Tip and SDB-RPS-Tip collectively contributed an additional 17.09% (Figure 2A). At the protein level, 1983 proteins (48.11% of the total) were shared among all methods, while the C18-Tip method uniquely identified 1155 proteins (28.02%). SCX-Tip and SDB-RPS-Tip each contributed fewer unique proteins (Figure 2B).

Correlation analysis indicated variable but generally strong concordance among the fractionation approaches (Figure 2C,D). For peptides, the correlation between C18-Tip and SCX-Tip was 0.67, and between C18-Tip and SDB-RPS-Tip, it was 0.65, whereas SCX-Tip and SDB-RPS-Tip exhibited a stronger correlation of 0.81 (Figure 2C). At the protein level, the correlations were generally higher: 0.81 between C18-Tip and SCX-Tip, 0.77 between C18-Tip and SDB-RPS-Tip, and 0.86 between SCX-Tip and SDB-RPS-Tip (Figure 2D). Together, these results demonstrate that combining multiple fractionation approaches, C18-Tip, SCX-Tip, and SDB-RPS-Tip, enhances spectral library coverage.

### 2.3. Comparative Analysis of DDA, DIA and BoxCar Methods

**Figure 3:**
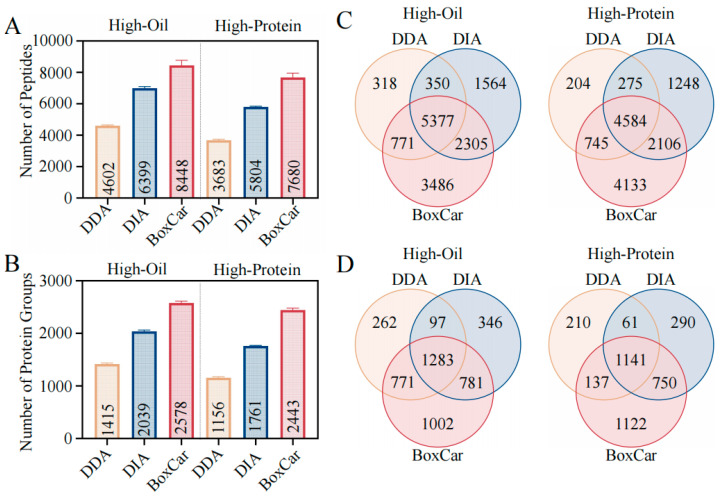
A comparative analysis of DDA, DIA, and BoxCar methods in High-Oil and High-Protein soybean groups. (**A**,**B**) The number of peptides (**A**) and proteins (**B**) identified by the DDA (yellow), DIA (blue), and BoxCar (red) methods in both High-Oil and High-Protein soybean groups. The data are presented as mean ± sd. (**C**,**D**) Venn diagrams illustrating the overlap of peptide (**C**) and protein (**D**) identifications among the DDA (yellow), DIA (blue), and BoxCar (red) methods in the High-Oil and High-Protein groups, highlighting unique and shared peptides and proteins detected by each method.

Using the spectral library as a reference, we compared the DDA, DIA, and BoxCar methods. In the High-Oil group, DDA identified 4602 peptides and 1415 proteins, while DIA and BoxCar identified 6993 peptides/2039 proteins and 8448 peptides/2578 proteins, respectively. In the High-Protein group, DDA identified 3683 peptides and 1155 proteins, while DIA and BoxCar identified 5804 peptides/1760 proteins and 7680 peptides/2442 proteins, respectively (Figure 3A,B). Notably, BoxCar yielded 82.2% more protein identifications in High-Oil seeds and 132.0% more in High-Protein seeds compared to DDA.

The Venn diagrams further highlight unique identifications by each method (Figure 3C,D). As illustrated in Figure 3C, DDA uniquely identifies 318 peptides (2.2% of the total) in the High-Oil group and 204 peptides (1.5% of the total) in the High-Protein group. After employing DIA and BoxCar methods, the percentage of specifically identified peptides in the High-Oil group increases to 11.0% (1564 of the total) and 24.5% (3486 of the total), respectively, while in the High-Protein group, it rises to 9.4% (1248 of the total) and 31.1% (4133 of the total) (Figure 3C). A similar pattern is observed at the protein level: DIA and BoxCar increase the proportions of uniquely identified proteins to 8.9% (346 of the total) and 25.7% (1002 of the total) in the High-Oil group and to 7.8% (290 of the total) and 30.2% (1122 of the total) in the High-Protein group (Figure 3D).

Overall, these results demonstrate that BoxCar markedly enhanced peptide and protein identifications in soybean seeds.

### 2.4. Evaluating the Reproducibility of DDA, DIA, and BoxCar Methods

**Figure 4:**
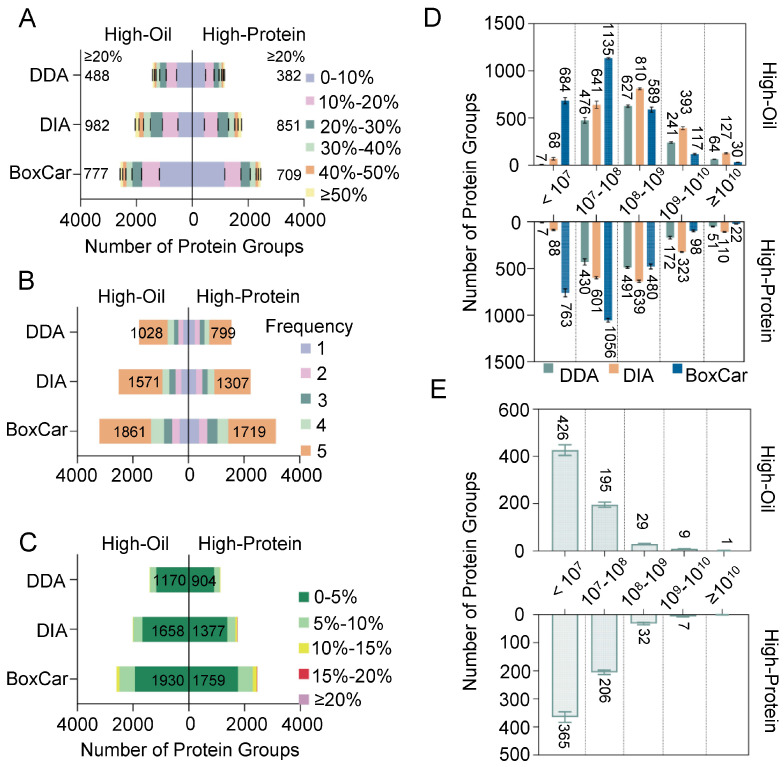
A reproducibility evaluation of the DDA, DIA, and BoxCar methods in High-Oil and High-Protein soybean groups. (**A**) Peptide coverage rates for proteins identified by the DDA, DIA, and BoxCar methods in both High-Oil and High-Protein groups, illustrating the depth of analysis provided by each method. (**B**) The frequency of protein identifications by the DDA, DIA, and BoxCar methods, showing the detection frequency of proteins across multiple samples in each group. (**C**) The distribution of coefficient of variation (CV) values for protein identifications by each method, assessing the consistency and reliability of protein quantification. (**D**) The distribution of protein abundances identified by each method across the High-Oil and High-Protein groups, providing insights into the range of protein concentrations detected. The data are presented as mean ± sd. (**E**) The distribution of protein abundances uniquely identified by the BoxCar method, demonstrating the extension of the detectable range for low-abundance proteins. The data are presented as mean ± sd.

To assess reproducibility, we examined the peptide sequence coverage and consistency of protein identification. In the High-Oil and High-Protein seeds, BoxCar identified 777 and 709 proteins, respectively, with >20% peptide coverage. In contrast, DDA identified 488 and 382 proteins above the same threshold, while DIA identified 982 and 851 proteins (Figure 4A; Table S5). Although DIA excelled in the >20% peptide coverage category, BoxCar captured more proteins in the 10–20% and <10% coverage ranges, suggesting superior detection in low-abundance regions (Figure 4E).

Statistical analyses of five replicate runs further underscore BoxCar’s consistency. In the High-Oil group, BoxCar identified 1861 proteins in all five replicates, while in the High-Protein group, 1719 proteins were consistently detected (Figure 4B; Table S5). DIA also exhibited good reproducibility (1571 proteins in the High-Oil group and 1307 in the High-Protein group across all replicates), surpassing DDA in both seed types.

Additionally, examining CV values revealed that BoxCar identified 1929 proteins in the High-Oil group and 1759 in the High-Protein group with CV < 5%. DIA identified 1658 and 1377 proteins, respectively, under the same CV threshold (Figure 4C; Table S5), whereas DDA identified only 1170 and 904 proteins. These outcomes confirm that BoxCar not only achieves greater depth but also higher reproducibility in soybean seed proteomics.

### 2.5. Evaluating the Abundance Range of Proteins Identified by DDA, DIA, and BoxCar Methods

Next, we evaluated the distribution of protein abundances identified by the DDA, DIA, and BoxCar methods. As illustrated in Figure 4D, BoxCar predominantly captured proteins with intensities below 10^9^, whereas high-abundance proteins (exceeding 10^9^) accounted for only 5.7% (147 of 2578) in the High-Oil group and 4.9% (120 of 2442) in the High-Protein group (Table S5). DIA, by contrast, displayed a more uniform distribution spanning 10^7^ to 10^10^, with high-abundance proteins comprising 25.5% (520 of 2039) in the High-Oil group and 24.6% (433 of 1760) in the High-Protein group. Similarly, DDA identified most proteins in the 10^7^ to 10^10^ range, with high-abundance proteins representing 21.6% (305 of 1415) in the High-Oil group and 19.3% (223 of 1155) in the High-Protein group. A further examination of proteins uniquely identified by BoxCar revealed that they predominantly clustered in the lower abundance region below 10^8^ (Figure 4E). These findings highlight the strength of the BoxCar method in detecting low-abundance proteins.

### 2.6. Application of BoxCar in Proteomic Analysis of Soybean Seeds

**Figure 5:**
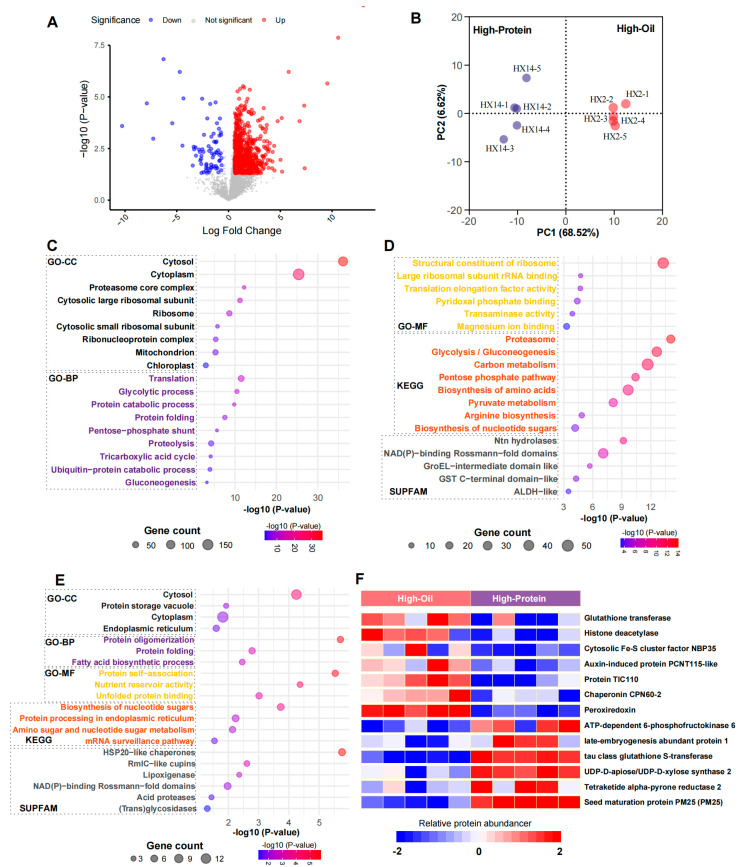
Proteomic differences between High-Oil and High-Protein soybean groups. (**A**) A volcano plot illustrating protein expression differences between the High-Oil and High-Protein groups. Proteins upregulated in the High-Oil group are marked in red (627 proteins), while those downregulated are shown in blue (72 proteins). *p*-values were calculated using a *t*-test. (**B**) The Principal Component Analysis (PCA) results demonstrating the two groups based on the differentially expressed proteins. (**C**,**D**) The enrichment results of signaling pathways for proteins upregulated in the High-Oil group. (**C**) shows the Gene Ontology Cellular Component (GO-CC, black) and Biological Process (GO-BP, pink) categories, while (**D**) displays Gene Ontology Molecular Function (GO-MF, yellow), KEGG pathways (orange), and SUPFAM domains (gray). (**E**) The pathway enrichment results for proteins upregulated in the High-Protein group. (**F**) A list of major specifically expressed proteins in both the High-Oil and High-Protein groups, highlighting the unique proteins that characterize each group.

The BoxCar method, with its ability to detect low-abundance proteins (Figure 4E), was used for an in-depth proteomic analysis of High-Oil and High-Protein soybean seeds. The comparative results revealed that the High-Oil group exhibited a substantially larger number of differentially expressed proteins, including 627 upregulated and 72 downregulated proteins compared to the High-Protein group (Figure 5A, Table S6). Among these, 46 proteins were exclusively detected in the High-Oil group, whereas 19 were unique to the High-Protein group (Table S6). The hierarchical clustering of these differentially expressed proteins clearly distinguished the two groups into separate clusters (Figure 5B).

Through pathway enrichment analysis, we discovered that the upregulated proteins in the High-Oil soybean seeds predominantly localized to the cytoplasm, proteasome, ribosome, and mitochondria (Figure 5C and Table S6). These proteins are involved in several key metabolic pathways, including glycolysis, gluconeogenesis, carbon metabolism, the pentose phosphate pathway, and amino acid biosynthesis (Figure 5C,D), suggesting that High-Oil seeds exhibit more robust metabolic processes. Furthermore, protein domain analysis revealed the significant enrichment of N-terminal nucleophile aminohydrolases, NAD(P)-binding Rossmann-fold domains, and GroEL domain like proteins (Figure 5D), highlighting the specialized biochemical attributes of High-Oil soybeans in protein folding, metabolic regulation, and energy conversion.

In contrast, only 72 proteins were upregulated in the High-Protein group, resulting in fewer enriched pathways. These proteins predominantly localized to the cytoplasm, protein storage vacuole, and endoplasmic reticulum (Figure 5E and Table S6) and are involved in processes such as nucleotide sugar biosynthesis, protein processing in the ER, protein oligomerization, fatty acid biosynthesis, and nutrient storage (Figure 5E). Domain analysis identified the enrichment of HSP20-like chaperones, RmlC-like cupins, lipoxygenases, acid proteases, and NAD(P)-binding Rossmann-fold domains (Figure 5E). Notably, the High-Protein group features protein storage vacuoles, although they appear to exhibit less dynamic metabolic activity compared to those in High-Oil seeds.

### 2.7. Major Difference Between High-Oil and High-Protein Groups

In total, 46 proteins were uniquely expressed in High-Oil seeds and 19 in High-Protein seeds (Table S6), highlighting distinct metabolic strategies employed by each seed type. Among the High-Oil-specific proteins, we identified 3 proteasome proteins, 8 chloroplastic/mitochondrial proteins, 11 cytoplasmic proteins, and 13 nucleic acid process-related proteins.

For example, cytoplasmic proteins such as glutathione transferase, histone deacetylase, cytosolic Fe-S cluster assembly factor NBP35, and auxin-induced protein PCNT115-like (Figure 5F) are involved in detoxification, gene expression regulation, energy metabolism, and hormonal signaling. These functions collectively support seed health and survival by mitigating oxidative stress, modulating gene expression in response to metabolic needs, and regulating energy production pathways critical for oil biosynthesis.

Additionally, chloroplastic and mitochondrial proteins including TIC110, peroxiredoxin, and chaperonin CPN60-2 (Figure 5F) are upregulated, enhancing photosynthetic capacity and antioxidative defenses. Enhanced photosynthesis provides the necessary energy and carbon skeletons for lipid synthesis, while antioxidative defenses protect the developing seeds from oxidative damage, thus promoting seed viability and developmental potential. Of particular note is GmTic110a, a critical chloroplast development protein whose mutation reduces chlorophyll levels and adversely affects photosynthesis, ultimately leading to fewer pods and a lower seed weight [32]. Peroxiredoxin contributes to maintaining oxidative homeostasis during seed maturation and dormancy, underscoring the importance of these subcellular proteins in High-Oil seeds.

By contrast, the High-Protein group primarily contained cytoplasmic proteins, including four key enzymes (Table S6). Notably, late embryogenesis abundant protein LEA-1, ATP-dependent 6-phosphofructokinase, and tau class glutathione S-transferase were upregulated, collectively improving stress tolerance, energy support, and metabolic efficiency. LEA proteins are known to protect cellular structures during desiccation and stress conditions, enhancing seed viability under adverse environmental conditions. ATP-dependent 6-phosphofructokinase plays a crucial role in glycolysis, providing energy and metabolic intermediates necessary for protein synthesis and accumulation.

Moreover, proteins such as UDP-D-apiose/UDP-D-xylose synthase 2, bifunctional dihydroflavonol 4-reductase/flavanone 4-reductase-like, and tetraketide alpha-pyrone reductase 2 (Figure 5F) play critical roles in reinforcing seed structural integrity, synthesizing defensive compounds, and bolstering chemical defenses. UDP-D-apiose/UDP-D-xylose synthase 2 is involved in cell wall biosynthesis, contributing to the structural robustness of seeds, while bifunctional dihydroflavonol 4-reductase/flavanone 4-reductase-like participates in flavonoid biosynthesis, which is essential for defense against pathogens and environmental stressors. Seed maturation protein PM25 offers additional biochemical and physiological protection during seed maturation, ensuring effective germination across various environmental conditions (Figure 5F).

Taken together, these findings demonstrate how High-Oil and High-Protein seeds employ distinct sets of proteins to optimize developmental processes, metabolic regulation, and environmental adaptation. High-Oil seeds enhance their metabolic capacity for oil biosynthesis and stress resistance, while High-Protein seeds focus on efficient protein accumulation and structural integrity to thrive in varying environmental conditions.

## 3. Discussion

This study demonstrates the clear advantages of the BoxCar method over the DDA and DIA methods in soybean proteomics. Notably, BoxCar significantly enhances sensitivity for detecting low-abundance proteins, advancing our understanding of the soybean proteome. This advantage could also extend to other crops with similarly wide dynamic ranges in protein content.

In particular, BoxCar achieved 82.2% and 132.0% more protein identifications in High-Oil and High-Protein seeds, respectively, compared to DDA. Similar improvements have been reported in urine proteomics [19]. Furthermore, BoxCar uniquely detected over 25.7% of proteins in High-Oil seeds (1002 proteins) and 30.2% in High-Protein seeds (1122 proteins), far exceeding the detection rates of DIA and DDA (Figure 3). Beyond improved identification depth, BoxCar also exhibits high reproducibility, with lower coefficients of variation (CVs) and more consistent results across replicates (Figure 4). The abundance distribution data confirm that BoxCar uniquely identifies proteins predominantly below 10^8 in intensity (Figure 4E), emphasizing its utility for low-abundance protein detection. These advancements provide deeper insights into the soybean proteome and have potential applications for proteomic analyses in other crops and tissues.

When comparing our findings to the existing literature, the enhanced sensitivity of BoxCar is not limited to soybean seeds. In Arabidopsis proteomics, BoxCar identified 8% more proteins than direct DIA and better quantified low-abundance proteins like kinases, phosphatases, and transcription factors [24]. BoxCar has also been applied successfully in several other Arabidopsis proteomic studies. For example, the loss of RVE8-like proteins in Arabidopsis led to altered carbohydrate, organic acid, and lipid metabolism, as well as a starch excess phenotype at dawn [31]. In another study, BoxCar technology quantified 6400 and 8500 protein groups in Arabidopsis seedlings under salt stress, respectively [32]. Similarly, in canola, BoxCar revealed that nutrient deficiency downregulated oxidative stress response proteins in roots, with calcium signaling playing a crucial role in the response [33].

In biomedical research, BoxCar has proven beneficial in diabetic urine proteomics, where it identified a larger proportion of low-abundance peptides (<10^7^ intensity) compared to DDA. BoxCar also significantly increased the number and sequence coverage of known diabetic biomarkers, including α-1-acid glycoprotein (ORM1/ORM2), α-2-macroglobulin (A2M), and β-2-microglobulin (B2M) [20]. In prostate cancer cell line proteomics, BoxCar showed improved protein identification depth and fewer missing values compared to DDA [34]. Moreover, BoxCar identified twice as many proteins in microvesicles from high-speed centrifugation, covering over 90% of DDA-identified proteins [35]. These result suggest that BoxCar is particularly effective for both deep proteome identification and the quantification of low-abundance proteins.

In our soybean seed proteomics, BoxCar revealed specific proteins and metabolic pathways associated with High-Oil and High-Protein traits (Figure 5). In High-Oil seeds, the upregulation of enzymes like TIC110 and peroxiredoxin highlights the importance of chloroplastic and mitochondrial pathways in oil biosynthesis and seed viability [34,35,36]. In High-Protein seeds, proteins such as tau class glutathione S-transferase and ATP-dependent 6-phosphofructokinase emerged as potential targets for optimizing protein accumulation [37,38].

When comparing our findings to the existing literature, it is clear that the enhanced sensitivity of BoxCar is not limited to soybean seeds. In the proteomic study of Arabidopsis, compared to direct DIA, BoxCar can not only identify 8% more proteins but also better quantify low-abundance proteins such as kinases, phosphatases, and transcription factors [24]. This suggests that BoxCar is effective in improving deep proteome identification and quantifying low-abundance proteins. BoxCar has already been applied in several studies in the field of plants. BoxCar proteomic analyses in Arabidopsis revealed that loss of RVE8-like proteins results in altered carbohydrate, organic acid, and lipid metabolism, including a starch excess phenotype at dawn [31]. Using BoxCar DIA technology, 6400 and 8500 protein groups were quantified from the shoots and roots of Arabidopsis seedlings under salt stress, respectively [32]. In addition, the quantitative proteomic analysis of canola based on BoxCar DIA technology revealed that nutrient deficiency led to the downregulation of oxidative stress response proteins in canola roots, with calcium signaling proteins playing a key role in the response of canola roots to nutrient deficiency [33].

In biomedical research, in a proteomic study of diabetic urine samples, the proportion of relatively low-abundance peptides (intensity < 10^7^) was larger in the BoxCar results compared to DDA, while BoxCar significantly increased the number and sequence coverage of seven previously reported potential diabetic biomarkers, including α-1-acid glycoprotein (ORM1/ORM2), α-2-macroglobulin (A2M), and β-2-microglobulin (B2M) [20]. In the study of proteins from the prostate cancer 3 cell line, BoxCar demonstrated improved protein identification depth and reduced missing values compared to the DDA method [34]. The number of proteins identified in the microvesicles obtained from high-speed centrifugation using the BoxCar method was twice that of DDA, covering over 90% of the proteins identified by DDA [35].

In the analysis of soybean seed differences, the comprehensive proteomic profiles enabled by BoxCar revealed specific proteins and metabolic pathways associated with High-Oil or High-Protein traits (Figure 5). For High-Oil seeds, the upregulation of enzymes such as TIC110 and peroxiredoxin underscores the importance of chloroplastic and mitochondrial pathways in oil biosynthesis and seed viability [34,35,36]. In High-Protein seeds, the elevation of proteins like tau class glutathione S-transferase and ATP-dependent 6-phosphofructokinase indicates potential molecular targets for optimizing protein accumulation [37,38].

In conclusion, BoxCar significantly enhances the detection of low-abundance proteins in soybean seeds, providing greater depth and accuracy in proteomic analyses. This improvement facilitates a deeper understanding of the molecular mechanisms driving High-Oil and High-Protein traits in soybean seeds and provides a powerful tool for advancing soybean seed biology and optimizing crop nutritional qualities.

## 4. Materials and Methods

### 4.1. Soybean Seed Collection and Protein Extraction

Huaxia-2 (HX2, High-Oil) and Huaxia-14 (HX14, High-Protein) soybeans were cultivated at the experimental farm of South China Agricultural University, located at 23°15′ N, 113°34′ E in Guangzhou, Guangdong Province, China. Mature soybean seeds were harvested approximately 70 days post planting.

To extract proteins, the harvested soybean seeds were first chopped into small pieces using a scalpel. The pieces were then rapidly frozen in liquid nitrogen to preserve cellular integrity and subsequently ground into a fine powder using a mortar and pestle. This powder was transferred into a 15 mL centrifuge tube, to which 10 mL of Tris-Mg/NP-40 buffer (0.5 M Tris-HCl, pH 8.3; 2% (*v*/*v*) NP-40; 20 mM MgCl_2_) was added. The mixture was subjected to ultrasonic disruption in a cell disrupter for 5 min, with an intermittent cycle of 2 s on and 2 s off, to ensure thorough cell lysis.

Following this, the lysate was centrifuged at 16,000× *g* for 20 min at 4 °C. The clear supernatant was carefully decanted into a new tube. To precipitate and remove high-abundance storage proteins, protamine sulfate was added to the supernatant to a final concentration of 0.05% (*w*/*v*). The mixture was incubated on ice for 30 min [30].

After incubation, the sample was centrifuged again at 16,000× *g* for 20 min at 4 °C. The supernatant was transferred to another new tube and mixed with four volumes of acetone and then left to precipitate proteins overnight at −20 °C. Following incubation, the mixture was centrifuged at 16,000× *g* for 10 min at 4 °C. The supernatant was discarded, and the precipitate was left to air-dry in a fume hood. The dry protein pellet was then re-solubilized in Tris-Mg/NP-40 buffer.

Finally, the protein concentration of the solution was quantified using the Bicinchoninic Acid (BCA) method [36], ensuring readiness for further analysis. This method ensures the efficient extraction of proteins from soybean seeds while minimizing protein degradation and contamination.

### 4.2. Peptide Preparation Using the FASP Method

For protein digestion, the Filter-Aided Sample Preparation (FASP) protocol was applied [25]. Begin by measuring 500 μg of protein and transferring it into a 30 kDa ultrafiltration tube. Centrifuge at 14,000× *g* for 15 min to remove any waste liquid. Subsequently, add 200 μL of 8 M urea (UA) solution, prepared with 100 mM Tris-HCl at pH 8.0, to wash the proteins.

Next, for the reduction and alkylation steps, add a solution containing 5 mM Tris(2-carboxyethyl)phosphine (TCEP) and 25 mM chloroacetamide (CAA). Allow the mixture to react at room temperature for 1 h. Following this, wash the proteins sequentially with 8 M UA and then with 50 mM ammonium bicarbonate (NH_4_HCO_3_), centrifuging after each wash to remove the previous solution.

After the washing steps, add trypsin at an enzyme-to-protein mass ratio of 1:20. Incubate the mixture at 37 °C for 16 h to facilitate enzymatic digestion. Once digestion was complete, centrifuge to collect the peptides from the filter. Dry the collected peptides in a vacuum centrifugal concentrator and store them at −80 °C until needed for LC-MS/MS analysis.

### 4.3. Peptide Fractionation Using C18-Tip, SCX-Tip and SDB-RPS-Tip Methods

**C18-Tip fractionation strategy**. Begin by weighing 10 mg of C18 beads and transferring them into a 100 μL tip lined with a C8 membrane. Rinse the C18-Tip three times with acetonitrile, ensuring complete filling during the final rinse, and allow it to stand at room temperature for 2 h. After incubation, centrifuge the tip to remove the acetonitrile. Next, wash the C18-Tip with NH_3_·H_2_O (pH = 10) to prepare for peptide loading. Transfer peptides dissolved in NH_3_·H_2_O (pH = 10) into the C18-Tip and centrifuge to discard the waste. Desalt the peptides with additional NH_3_·H_2_O (pH = 10). Elute the peptides using a gradient of acetonitrile in NH_3_·H_2_O (pH = 10) at increasing concentrations (6%, 9%, 12%, 15%, 18%, 21%, 25%, 30%, 35%, and 50%).

**SCX-Tip fractionation strategy**. First, dissolve the peptide sample in 1% trifluoroacetic acid (TFA) and transfer it to a 100 μL Tip column equipped with five layers of SCX membrane. After centrifugation to remove waste, wash the peptides with 0.2% TFA. Sequentially elute the peptides with solutions containing ammonium acetate (AA) in 20% acetonitrile (ACN) and 0.5% formic acid (FA), at concentrations of 50 mM, 75 mM, 125 mM, 200 mM, and 300 mM, followed by a final elution with 80% ACN and 5% NH_3_·H_2_O. Dry the eluted peptides in a vacuum centrifugal concentrator and store them at −80 °C for future LC-MS/MS analysis.

**SDB-RPS-Tip fractionation strategy.** Start by assembling five layers of SDB-RPS membranes into a 100 μL Tip column. Dissolve the peptides in 0.1% TFA and load them onto the SDB-RPS-Tip column. After centrifugation to eliminate waste liquid, perform a washing step with 0.2% TFA. Sequentially elute the peptides with increasing concentrations of ammonium formate (AF) in ACN and 0.5% FA (100 mM AF/40% ACN, 150 mM AF/60% ACN), followed by a final elution with 80% ACN and 5% NH_3_·H_2_O. Heat and dry the peptides in a vacuum centrifugal concentrator before storing them at −80 °C for subsequent LC-MS/MS analysis.

### 4.4. LC-MS/MS Analysis

All samples were analyzed using an EASY-nLC 1200 system coupled to an Orbitrap Fusion Lumos Tribrid mass spectrometer (Thermo Fisher Scientific, San Jose, CA, USA). The peptides were separated on a homemade C18 column (100 μm ID × 30 cm length). The mobile phases consisted of 0.1% formic acid in water (Phase A) and 0.1% formic acid in 80% acetonitrile (Phase B). Peptides were eluted at a flow rate of 600 nL/min using a linear gradient of acetonitrile in 0.1% formic acid over 78 min, with the gradient schedule as follows: 0–5 min (4–10% B), 5–48 min (10–22% B), 48–66 min (22–35% B), 66–76 min (35–90% B), and 76–78 min (90% B).

**DDA MS acquisition strategy.** MS and MS/MS scans were conducted using an Orbitrap mass analyzer (Thermo Fisher Scientific, San Jose, CA, USA). Full MS scans covered a range of 350–1550 *m*/*z* at a resolution of 120,000. The automatic gain control (AGC) target was set at 4e^5^ with a maximum ion injection time (MIT) of 50 ms for full scans. Precursor ions selected for MS/MS had an isolation width of 1.6 *m*/*z* and were fragmented at a normalized collision energy of 30%. MS^2^ resolution was set at 15,000, with an AGC target of 5e^4^ and an MIT of 30 ms for MS/MS scans. Dynamic exclusion was set for 18 s.

**DIA MS acquisition strategy.** Similar to DDA, the full scan range was 350–1550 *m*/*z* at a resolution of 120,000. AGC was set to 4e^5^ with an MIT of 60 ms for full scans. MS^2^ scans were performed at a higher resolution of 30,000, with an AGC target of 5e^4^ and an MIT of 54 ms. Stepped collision energy was configured at 27 (±3%). Thirty variable DIA windows spanned from 350 to 1550 *m*/*z* (Table S7).

**BoxCar MS acquisition strategy.** The full scan range was extended to 350–1650 *m*/*z* at a high resolution of 240,000. AGC was set at 1e^6^ for full MS scans, with an automatic MIT. The cycle included three BoxCar scans using 10 boxes each (1 Th overlap) (Table S7). Precursor ions for MS/MS were isolated with a width of 1.4 *m*/*z* and fragmented using a normalized collision energy of 27%. MS2 resolution remained at 15,000, with an AGC target of 5e^4^ and dynamic MIT settings. Dynamic exclusion lasted for 30 s.

### 4.5. Data Analysis

All MS raw files were analyzed using MaxQuant [37,38] software, version 2.0.3.0. Peptide lists were searched against the species-level Uniprot TrEMBL&Swiss-Prot Glycine max database [39]. Protein N-terminal acetylation and methionine oxidation were set as variable modifications, while the carbamidomethylation of cysteine was set as a fixed modification. The maximum number of allowed missing cleavage sites was set to two. Precursor spectra were searched with an accuracy of 4.5 ppm, and fragment spectra were searched with an accuracy of 20 ppm.

Protein data preprocessing and differential analysis were performed using Perseus [40]. Missing values were imputed in Perseus using a normal distribution (width: 0.3, downshift: 1.8, mode: separately for each column). Proteomes of High-Oil and High-Protein soybean seeds were analyzed for differential expression using a *t*-test. Proteins with |log2(fold change)| ≥ 1.5 and *p*-value ≤ 0.05 were defined as significantly differentially expressed genes (DGEs). Gene Ontology (GO) function and Kyoto Encyclopedia of Genes and Genomes (KEGG) pathway enrichment analyses for DEGs were performed using DAVID 2021 (Dec. 2021). Specifically expressed proteins in the High-Oil and High-Protein groups were annotated using the DAVID database. GraphPad Prism software (version 9.5.1) was used for data visualization.

## Data Availability

The datasets supporting the conclusions of this study are included within the article and its Supplementary Materials. The raw proteomic data files, stored in the thermo.raw format, are accessible through the iProX database [41,42]. These files can be found in the iProX database under the accession number PXD052278.

## Supplementary Materials

The following supporting information can be downloaded at: https://www.mdpi.com/article/10.3390/ijms26030949/s1.
